# Unsupervised Learning Applied to the Stratification of Preterm Birth Risk in Brazil with Socioeconomic Data

**DOI:** 10.3390/ijerph19095596

**Published:** 2022-05-05

**Authors:** Márcio L. B. Lopes, Raquel de M. Barbosa, Marcelo A. C. Fernandes

**Affiliations:** 1 Laboratory of Machine Learning and Intelligent Instrumentation, Federal University of Rio Grande do Norte, Natal 59078-970, Brazil; marciojunior159@gmail.com; 2 Department of Pharmacy and Pharmaceutical Technology, University of Granada, 18071 Granada, Spain; rbarbosa@ugr.es; 3 Department of Computer Engineering and Automation, Federal University of Rio Grande do Norte, Natal 59078-970, Brazil

**Keywords:** preterm birth, clustering, unsupervised learning, PTB risk, Brazil

## Abstract

Preterm birth (PTB) is a phenomenon that brings risks and challenges for the survival of the newborn child. Despite many advances in research, not all the causes of PTB are already clear. It is understood that PTB risk is multi-factorial and can also be associated with socioeconomic factors. Thereby, this article seeks to use unsupervised learning techniques to stratify PTB risk in Brazil using only socioeconomic data. Through the use of datasets made publicly available by the Federal Government of Brazil, a new dataset was generated with municipality-level socioeconomic data and a PTB occurrence rate. This dataset was processed using various unsupervised learning techniques, such as *k*-means, principal component analysis (PCA), and density-based spatial clustering of applications with noise (DBSCAN). After validation, four clusters with high levels of PTB occurrence were discovered, as well as three with low levels. The clusters with high PTB were comprised mostly of municipalities with lower levels of education, worse quality of public services—such as basic sanitation and garbage collection—and a less white population. The regional distribution of the clusters was also observed, with clusters of high PTB located mostly in the North and Northeast regions of Brazil. The results indicate a positive influence of the quality of life and the offer of public services on the reduction in PTB risk.

## 1. Introduction

Complications of preterm birth (PTB), defined as a birth which happens prior to the 37th week of pregnancy, are the most common cause of mortality among children of 5 years old or younger [1,2,3]. In addition, it was shown as a critical factor for the survival of newborns [2]. Preterm-born babies present a major challenge to medical assistance, which needs to supplement their yet not fully developed vital organs [4]. Trying to understand and to prevent the causes of PTB has become increasingly more common in scientific research, especially with the recent emergence of progressively more trustful and more complex government-owned datasets. Coming closer to this goal could mean finding of ways of preventing PTB or at least of anticipating it, thus providing assistance to the mother in time, possibly reducing the amount of lives lost.

The work presented in [5] shows that PTB’s etiology is multi-factorial and that the risk of PTB could be associated with the socioeconomic situation of a given region (neighbourhood socioeconomic status or neighbourhood SES). Neighbourhood SES is an area-level measurement which aggregates SES factors (such as income, education and employment) in a certain geographic level [6]. Research shows that PTB rates are higher in areas with low SES when compared to areas with high SES [7].

Numerous machine learning techniques have been previously applied to the problem of PTB prediction or stratification, including SVMs [8], neural networks [9,10,11] and decision trees [12,13]. However, the most commonly applied techniques are logistic regression and linear regression, employed in the analysis and prediction of PTB for various factors: poverty [14], pregnant mother’s working conditions [15,16], general social factors [17,18] and, mainly, clinical and hereditary factors [19,20,21,22]. There is a vast literature associating different factors to preterm birth using traditional statistical methods [23,24,25], including an association of social factors [26,27].

One way of comprehending the associated risk of the many SES factors to the occurrence of PTB is through data clustering. Clustering is a segment of unsupervised machine learning techniques that seek to associate and group elements together without any initial comprehension of the data themselves. In order to do so, clustering techniques make use of distancing algorithms to judge how close or similar two points are from each other and whether or not they should be grouped in the same cluster. Clustering techniques have been used for scientific analyses for decades in many areas, such as psychology [28], genetics [29] or geophysics [30].

Clustering as the method to discover the groups which are more vulnerable to PTB risk is less common than traditional statistical methods. However, its application can already be seen in some recent studies. In [31], spacial clustering shows a possible relation between living closer to landfills and PTB occurrences. The studies presented in [32,33] show the clustering of hereditary and behavioural factors associating them with PTB risk. In addition, in [34], the study investigates the geographical distribution of PTB risk in Paris by clustering at the level of “census blocks”.

Thereby, the main objective of this work is to stratify the risk of PTB in Brazil from SES factors in order to confirm or deny that PTB occurrence is indeed related to socioeconomic conditions, a common general finding of multiple previous works in this area. The stratification process is conducted through clustering analysis based on unsupervised machine learning techniques. The analysis was performed by combining three freely available datasets collected by the Federal Government of Brazil: *Sistema de Informações sobre Nascidos Vivos* (SINASC) [35], containing data regarding gestation, birth, newborns and mothers; *Cadastro Único* (CADU) [36], containing a wide range of socioeconomic data from Brazilian citizens on a personal and family level; and the population estimate as disclosed by *Instituto Brasileiro de Geografia e Estatística* (IBGE) [37]. A new dataset was generated from the combination of these datasets and a new metric—PTB Municipal Rate (PMR)—was created. These two were used together in a clustering analysis, at municipal level, seeking to visualise the relation between SES factors and PTB risk. This article presents an analysis of some SES factors associating them with each discovered cluster. That way, the results presented in this work might contribute to the elaboration of more efficient and specialised politics for the Brazilian public health service.

## 2. State of the Art

The relationship between SES factors and occurrence of PTB, as mentioned before, has been studied by many authors, mostly but not exclusively dealing with just one or two “dimensions” of SES (e.g., education and income) and traditional statistical comparison methods rather than machine learning. For instance, this is the case observed by [16], where working conditions are observed together with preterm birth. Women with long work-hour schedules and those reportedly dissatisfied with their current work are shown to have significant higher risk of PTB in European countries. Women working excessively long hours (over 43 h/week) were found to have a preterm delivery odds ratio of 1.33 compared the unity (30–39 h/week), and women who had to work in standing position for over 6 h had an odds ratio of 1.26 compared to the unity (less than 2 h). These findings put working conditions and stressful situations as some of the possible non-biological factors to influence PTB, a view also strengthened by the results observed by [38], whose study observes the same relation of work, stress and PTB in Cypriot women.

The possibility of a certain region’s sanitation and housing conditions affect birth delivery time is explored by related recent studies by [26,39,40]. These studies present access to proper sanitation facilities as possibly an important factor to help increase PTB occurrences among Indian women. All studies obtained a statistically significant difference in the frequency of PTB outcomes when contrasting people with toilet access with people with no toilet access. Furthermore, the results of [26] also suggest that the harassment of girls and women (stressful event) and excessive time fetching water (over 2 h/day, manual labour) increase the risk of PTB, with odds ratios of 1.26 and 1.33, respectively. The results of [39,40] also include analyses on education data, and, in both studies, women with higher levels of education appear to have significantly lower risk of PTB.

Education as a social factor that raises the risk of PTB is also defended by the meta-analysis presented by [27]. The analysis was performed over 12 distinct countries’ groups of mothers, collected in different years and using different education indicators. Its results indicate that mothers with low levels of education are more likely to experience PTB, with an increased risk of 48% and 84% on the two scoring methods used. This is a considerably large difference and is a strong indication that education is an important aspect when exploring PTB factors. The same idea is given by the study provided by [41], where the higher educated women in Lombardy are shown to have 19% less risk of experiencing PTB, and a reduced risk was also observed when analysing foreign-born and local-born mothers separately.

The notion of relating all or most of these social factors at once and studying and treating them all as factors of social deprivation or social inequality is seen with association to preterm in the study presented in [5]. The study merges these factors into an SES Neighbourhood feature and associates it with personal data from the patients. The results given by intra-cluster correlation indicate SES neighbourhood-level circumstances to be responsible for 5.72% of all variance in PTB. Although only a small portion of the total variance, this can have considerable impact on model fine-tuning if one aims to develop a preterm predictor, and it provides a strong case for the continued studies on socioeconomic factors and PTB.

Another study to tackle the relationship of SES Neighbourhood and preterm was presented by [42] and also had results that advocate for the importance of the socioeconomic environment to PTB. The main difference of this study when compared to [5] is that their study used income variation over time as a way of measuring the socioeconomic status of neighbourhoods, with this different method obtaining final numbers that showed that women living in areas of low socioeconomic indices or in areas where socioeconomic levels are declining have higher risk of PTB occurrence. Stable Low-level areas (i.e., low-level areas that do not show progress in socioeconomic factors) had the highest odds ratio of 1.20—compared to Stable High-level areas.

A recent study by [34] also uses SES Neighbourhood to investigate preterm birth across the city of Paris’ block areas using spatial clustering, and its results endorse the idea of SES factors as an influential factor of PTB. When using SES Neighbourhood as cluster detection variables, the clustering resulted in a final cluster division with a *p*-value of 0.06, but when adjusting for SES, removing it from the clustering, the *p*-value increased to 0.81, a much less significant number, indicating that SES Neighbourhood was responsible for a great portion of the explainable PTB variance.

As it has already been put, most of these works presented above, as well as most of the non-cited related literature, make use of traditional statistical methods, associating a selected range of features and verifying possible correlations. The three latest mentioned works [5,34,42], go one step further and work with a merged value of many dimensions, but they still need a subjective human decision on how to unite these values into a significant feature. A few questions yet unanswered or only partially answered on PTB and SES are as follows: (1) If such a relationship exists and is significant, can high and low PTB areas be discovered through the clustering of SES factors? (2) Is this relationship intrinsic enough that it can be found automatically by a machine without any significant feature selection? (3) Is it possible to uncover the socioeconomically deprived areas most likely to suffer from high PTB numbers? (4) Which SES factors are more likely to alter considerably in regions with high and low PTB occurrences? Therefore, the current work contributes to the research area by attempting to fully or partially answer these questions by using two distinct unsupervised learning methods to explore large Brazilian datasets of SES and birth data.

By combining *k*-Means and DBSCAN, two very different clustering algorithms, the first method also contributes by creating a new method for targeted cluster analysis. The algorithm initially provides a free clustering layer of *k*-Means clustering, with results then filtered by a target variable excluded from the initial cluster. The results are then passed to a final/decision cluster, generalising and removing clusters to provide the final results. This method allows us to completely isolate PTB from the SES clustering, while also finding significant clusters without having to rely a traditional optimal cluster techniques, which would ignore the external targeted variable.

## 3. Materials and Methods

### 3.1. Datasets

As described in the introduction, three datasets were used to generate the training set: SINASC, CADU and IBGE. The analysis performed in this work dealt only with data from 2018 for all the datasets used.

The SINASC dataset, here characterised by the variable $T_{\mathrm{SN}}$, is a dataset with 61 features and almost 3 million samples. It stores data related to births that occurred on Brazilian territory, and it can be found at the DATASUS website [35]. For the purpose of this work, two columns of $T_{\mathrm{SN}}$ were used, one related to the gestational period length and the other to the mother’s municipality of residence, as shown in Table 1.

The CADU dataset was split into two distinct datasets: CADU Individual, characterised by the variable $T_{P}$, and CADU Household, characterised by $T_{F}$.

The $T_{P}$ dataset has over 12 million samples of Brazilian citizens, each with 26 features, storing basic individual data, such as gender, age, and race, but also more specific data about education and employment, as described in Table 2.

In the $T_{F}$ dataset, there are about 4 million household samples, each with 23 features, including data about living conditions, household income and type of family, as detailed in Table 3.

The population dataset used here is the official 2018 population estimate dataset by IBGE, which includes estimates for 5570 municipalities. Represented here by $T_{\mathrm{IBGE}}$, this dataset has 5 columns, described in Table 4, of which only 2, referring to total population and municipality code, were used.

The sources and dimensions of each dataset for the year 2018 can be seen in Table 5.

### 3.2. Preprocessing

**Figure 1:**
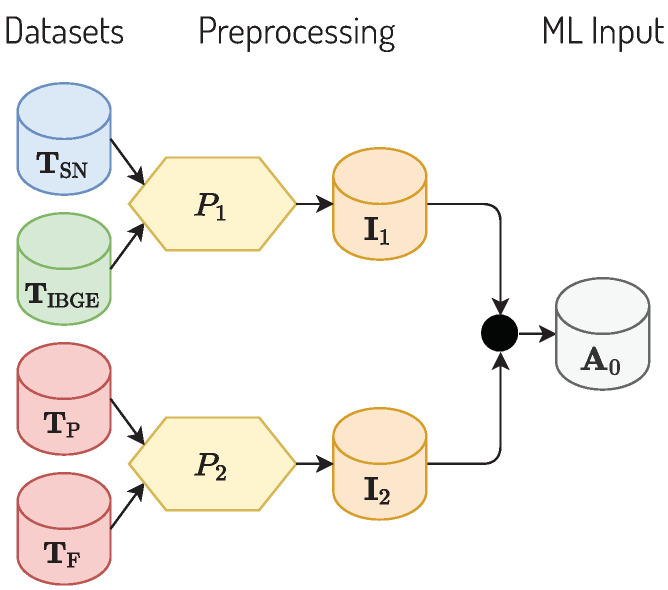
General preprocessing scheme to generate dataset $A_{0}$ (machine learning process input), including preprocessing stages $P_{1}$ and $P_{2}$, their outputs $I_{1}$ and $I_{2}$ and the original datasets $T_{\mathrm{SN}}$, $T_{\mathrm{IBGE}}$, $T_{P}$ and $T_{F}$.

In order to join the information available in all four datasets so it can serve as the input for a clustering algorithm, a preprocessing stage was designed, being subdivided into: preprocessing $P_{1}$, dealing with $T_{\mathrm{SN}}$ and $T_{\mathrm{IBGE}}$, and preprocessing $P_{2}$, dealing with $T_{P}$ and $T_{F}$. $P_{1}$ and $P_{2}$ generate, respectively, intermediate datasets $I_{1}$ and $I_{2}$ as output. These intermediate datasets are composed of 5570 samples each, one for each Brazilian municipality, and they both are joined together using the unique Municipality Code, generating $A_{0}$. Figure 1 details the general preprocessing scheme.

#### 3.2.1. Preprocessing $P_{1}$

As described in Figure 1, the SINASC dataset, characterised by the variable $T_{\mathrm{SN}}$, was preprocessed to obtain the number of preterm births by municipality.

First of all, $T_{\mathrm{SN}}$ was filtered by weeks of pregnancy, keeping only the samples with less than 37 weeks. Then, $T_{\mathrm{SN}}$ was grouped by the mother’s residential municipality code, counting the number of samples for each municipality.

Next, the grouped $T_{\mathrm{SN}}$ was joined with the population estimate dataset ($T_{\mathrm{IBGE}}$) through the Municipality Code. As described in Figure 1, the SINASC dataset, characterised by the $T_{\mathrm{SN}}$ variable, was preprocessed to obtain the total number of PTB occurrences by municipality.

First, $T_{\mathrm{SN}}$ was filtered by weeks of gestation, keeping only those samples with less than 37 weeks. Next, $T_{\mathrm{SN}}$ was grouped by the mother’s municipality of residence, counting how many occurrences were registered in each municipality.

Then, by joining $T_{\mathrm{SN}}$ with the IBGE population estimate dataset ($T_{\mathrm{IBGE}}$), using the Municipality Codes present in both datasets, the information about population size was added to $T_{\mathrm{SN}}$, generating intermediate dataset $I_{1}$.

On $I_{1}$, it was then calculated the PTB Municipality Rate PMR. PMR is the metric proposed in this work to measure the frequency of PTB occurrences by municipality, expressed as the following:

(1) $$\mathrm{PMR}=\frac{N_{\mathrm{NP}}}{N_{P}}$$

where $N_{\mathrm{NP}}$ is the total number of PTB occurrences in a given municipality, and $N_{P}$ is the population of that same municipality.

During the research, it was decided to use the total population instead of the total number of births—which could be obtained from $T_{\mathrm{SN}}$—because it was observed that some of the PTB over total number of births fractions were resulting in very unrealistic percentages: for instance, one municipality had 70% of its registered births reported as PTB. Even though the most extreme values will be removed later in process $P_{3}$, the total number of births was replaced by the population so that the possibility of these unbalanced values impacting municipalities of closer-to-the-median PMR may reduce, assuming that $T_{\mathrm{SN}}$ is missing data.

The output of preprocessing $P_{1}$ is the intermediate dataset $I_{1}$, comprised of 2 columns: Municipality Code and PMR.

#### 3.2.2. Preprocessing $P_{2}$

As described in Figure 1, datasets CADU Individual, expressed by variable $T_{P}$, and CADU Household, characterised by variable $T_{F}$, were preprocessed in order to turn their categorical features into numerical features, able to be used by the selected clustering algorithms.

First, the dataset $T_{P}$ was filtered, removing all samples of people who are male and also female under 14 or over 40 years of age, removing ages of recognisable less fertility [43]. Next, one-hot encoding was applied to all the categorical features, generating 29 new binary features. Finally, an additional processing was to aggregate educational features representing the same educational level. As for the $T_{F}$ dataset, which represents data on a family level, one-hot encoding was applied to all categorical features, generating 48 new binary features. The datasets were joined by the Household ID, present in both, adding household data present in $T_{F}$ to each person sample of $T_{P}$ that belongs to that household. At the end of the process, values were grouped by municipality of residence, calculating each feature’s average by municipality. Thus, the output of $P_{2}$ was the intermediate dataset $I_{2}$.

#### 3.2.3. Output $A_{0}$

In order to generate the general preprocessing output, indicated by the dataset $A_{0}$, the outputs of $P_{1}$ and $P_{2}$ (datasets $I_{1}$ and $I_{2}$, respectively) were joined by Municipality Code. Dataset $A_{0}$ is comprised of 5529 samples and 104 features, and each sample represents a Brazilian municipality.

### 3.3. Methodology

**Figure 2:**
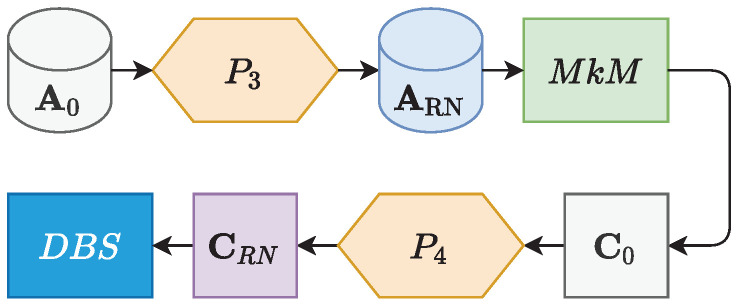
Clustering process sequence diagram, including both employed algorithms MkM and DBS, preprocessing stages $P_{3}$ and $P_{4}$, and intermediate datasets generated in each stage.

The methodology used for generating the final clusters followed the sequence of stages seen in the diagram of Figure 2. In which $A_{0}$ was turned into a intermediate dataset $A_{\mathrm{RN}}$ through the $P_{3}$ preprocessing. Then, $A_{\mathrm{RN}}$ was processed by a clustering block called multiple *k*-means, identified here as MkM. MkM generates a centroids matrix, here referenced by the variable $C_{0}$. $C_{0}$ then went through the last preprocessing stage $P_{4}$, generating a new matrix $C_{\mathrm{RN}}$. Finally, $C_{\mathrm{RN}}$ was processed by the density-based spatial clustering of applications with noise (DBSCAN) algorithm, where the final clusters were revealed. The DBSCAN model is referenced here as DBS.

#### 3.3.1. Preprocessing $P_{3}$

**Figure 3:**
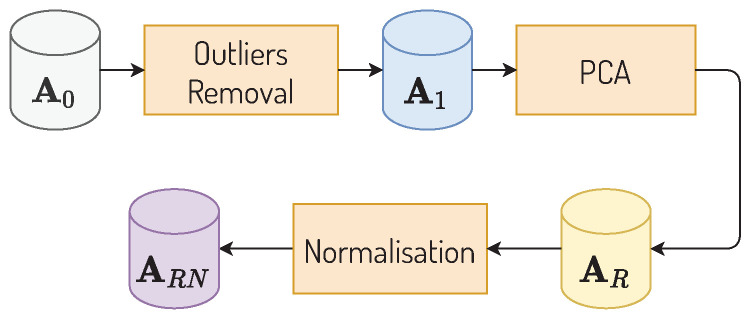
$P_{3}$ preprocessing diagram.

As shown in Figure 3, the $P_{3}$ preprocessing is characterised by removal of outliers, dimensionality reduction and normalisation. First, municipalities considered as PMR outliers, that is, those whose PMR values are three standard deviations above or below the national average, were removed from $A_{0}$, generating a new dataset $A_{1}$. The column that stores PMR values was removed from $A_{1}$, and its data was stored separately for future uses. $A_{1}$ is comprised of 103 features, and considering the natural difficulty of optimising clusters in high dimensions (curse of dimensionality), principal component analysis (PCA) was used to reduce dimensions, generating a reduced dataset $A_{R}$ that kept 95% of $A_{1}$’s variance with 58 features. Next, $A_{R}$ was normalised through the cascading application of 3 techniques: (1) Yeo–Johnson Transformation, making the original dimensions’ distribution more normal distribution-like; (2) L2 sample normalisation, re-balancing samples individually to capture points of higher and lower impact in each; (3) and finally, 0 to 1 normalisation by feature. These normalisation techniques generate the *k*-means (MkM) input dataset $A_{RN}$.

#### 3.3.2. Multiple *k*-Means (MkM)

In order to find representative centroids associated to our problem, a processing strategy, here called MkM, was proposed. MkM is characterised by a group of *N k*-means models that are all executed to the same input: $A_{RN}$. Each *i*th *k*-means model, here called $kM_{i}$, is executed with a determined number of centres, $Nc_{i}$, randomly initialised. At the end, they generate a matrix expressed as:

(2) $$C_{0}=\left[\begin{array}{c} G_{1}^{T}\\ \vdots \\ G_{N}^{T} \end{array}\right]$$

where each *i*th $G_{i}$ is characterised by a set of $Nc_{i}$ centres associated to each *i*-th $kM_{i}$ model, and they are expressed as:

(3) $$G_{i}=\left[c_{i,1},\cdots ,c_{i,Nc_{i}}\right]$$

where $c_{i,j}$ is the *j*-th centre of the *i*-th $kM_{i}$ model, and they are expressed as:

(4) $$c_{i,j}=\left[c_{i,j,1},\cdots ,c_{i,j,H}\right]$$

where *H* represents the total number of features from the inputted dataset, which, in this work, equals H=58, as mentioned in the past subsection. So, the matrix $C_{0}$ can be rewritten as:

(5) $$C_{0}=\left[\begin{array}{c} c_{1,1}\\ \vdots \\ c_{1,Nc_{1}}\\ \vdots \\ c_{N,1}\\ \vdots \\ c_{N,Nc_{N}} \end{array}\right]=\left[\begin{array}{ccc} c_{1,1,1} & \cdots & c_{1,1,H}\\ \vdots & \ddots & \vdots \\ c_{1,Nc_{1},1} & \cdots & c_{1,Nc_{1},H}\\ \vdots & \ddots & \vdots \\ c_{N,1,1} & \cdots & c_{N,1,H}\\ \vdots & \ddots & \vdots \\ c_{N,Nc_{N},1} & \cdots & c_{N,Nc_{N},H} \end{array}\right].$$

The number of rows in $C_{0}$ can be determined as:

(6) $$L=\sum_{i=1}^{N}Nc_{i}.$$

For this work, N=290 was used, that is, 290 *k*-means models were executed for $A_{RN}$. The number of centres in *k*-means varied from 2 to 30, that is, $Nc_{i}\in \left\{2,\cdots ,30\right\}$ (29 different numbers), and for each number of centres, 10 different instances of the model were run, adding up to N=290 models. Each *i*-th model, $kM_{i}$, was optimised with the expectation-maximisation algorithm, the tolerance for convergence was set to $10^{-7}$, and the maximum number of iterations was 10,000. The results from each of the N=290 models were in a matrix $C_{0}$ with L=4640 samples, representing all the centres detected by the models.

In addition $C_{0}$, a new intermediate dataset $A_{C}$ is created, comprised of 5529 samples and N+1 features. Each sample represents a municipality, and the features are the municipality’s PMR and the clusters each sample is associated with for each *n*-th *k*-means model found in MkM. Thereby, each municipality, in each *i*-th row, belongs to a *k*-th cluster in each *n*-th column of $A_{C}$. Each *n*-th *k*-means model generated by MkM has $N_{c}$ clusters grouping a set of *B* municipalities.

#### 3.3.3. Preprocessing $P_{4}$

In preprocessing $P_{4}$, matrix $C_{0}$ was filtered, keeping only the centres that represent clusters treated as “Clusters of Interest” (CoI), that is, those clusters whose mean PMR exceeds or is exceeded by the national average PMR in at least 10%. The data associated to each cluster are obtained through $A_{C}$, and the mean PMR can be calculated as:

(7) $$TMP_{\mathrm{media}}=\frac{1}{B}\sum_{i=1}^{B}TMP_{i}$$

where $TMP_{i}$ is the PMR related to the *i*-th municipality, and *B* is the total number of municipalities for a given cluster. For the calculation of the national PMR, the formula is used considering the cluster of municipalities to be one containing all the municipalities present in the datasets and *B* to be the total number of municipalities: in this case, B=5529. After filtering $C_{0}$, a new matrix is generated containing containing only the centres of the CoI, here referred to as $C_{ci}$.

In order to group the CoI, $C_{ci}$, retrieved from the multiple *k*-means executions by the MkM block, a correlation matrix was generated from the samples of $C_{0}$. The idea here is to work with the similarity between the centres—representing the CoI—to facilitate the clustering process in the following stages. The correlation matrix is characterised by the variable $C_{1}$.

**Figure 4:**
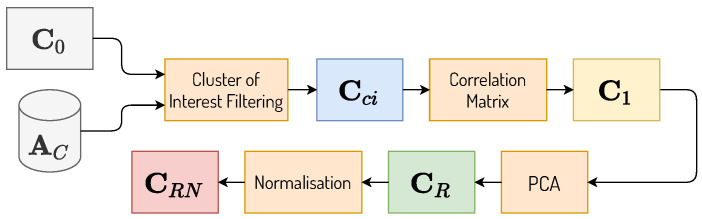
$P_{4}$ preprocessing diagram.

In order to reduce the dimensionality of the correlation matrix $C_{1}$, the PCA algorithm was a applied to $C_{1}$, generating a reduced matrix $C_{R}$. $C_{R}$ is composed of 4 columns that maintain 99% of the original variance of $C_{1}$. Next, $C_{R}$ was normalised by column to keep values between 0 and 1, generating $C_{RN}$. Figure 4 details the $P_{4}$ preprocessing.

#### 3.3.4. DBSCAN (DBS)

The DBSCAN receives $C_{RN}$ as input, containing the sample correlation data. Unlike *k*-means, DBSCAN does not need a fixed target number of centres (clusters) to be set beforehand because its functionality is defined solely by the adjustments in two parameters: minimum Euclidean distance between two points (ϵ) and minimum amount of points per cluster. As output, DBS generates a classification of the clusters discovered by the MkM from their respective centres, grouping together those so similar they can be treated as different instances of the same cluster, while also discarding centres without enough similar pairs and treating these as noise.

For DBS, the minimum number of occurrences was defined as 50, and ϵ=0.06. As there is not a well-defined or standard method to measure the quality of the validated clusters, data visualisation techniques were used to verify cluster consistency. Graphical representations were created to show the municipality-cluster association and also to visualise $C_{RN}$ post-classification (2D plot with t-SNE). The parameters were adjusted in order to avoid too much regional fragmentation (clusters with no regional aspect, over-fitting) or empty regions (no classified municipalities in a large map area, under-fitting).

### 3.4. Computational Tools

In order to achieve the goals of this research, we made use of a few Python implementations of the aforementioned algorithms, designed by the Scikit-Learn [44] and Pandas [45,46] projects. The exact way each of them was used within our workflow can be thoroughly observed in an online repository [47]. All the main scripts developed for this research were made publicly available in the repository, as well as some of the obtained datasets, including the one representing dataset $A_{0}$.

## 4. Results

**Figure 5:**
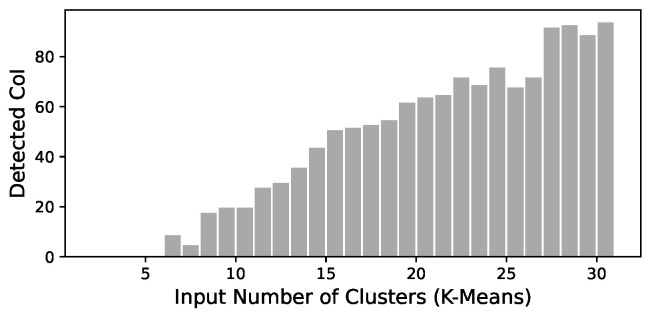
Clusters discovered by MkM by input number of clusters.

After the application of $P_{1}$, $P_{2}$ and $P_{3}$, the MkM model generated a total of 1337 CoI. The number of detected CoIs grew according to the total numbers of clusters defined in the k-Means models, $Nc_{i}$, as it can be seen in Figure 5. It also shows that the first cases of CoI appear when the *k*-means input number of centres, $Nc_{i}$, equals 5, reaching about 90 CoIs for the highest number of $Nc_{i}$ (27 to 30).

**Figure 6:**
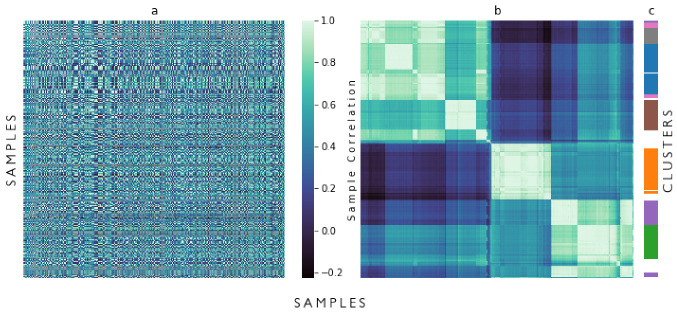
(**a**) Correlation matrix, (**b**) Correlation Matrix reordered by distance between samples, and (**c**) Classification of reordered samples after DBSCAN.

The correlation matrix, $C_{1}$, and its reordered version can be seen in items (a) and (b) of Figure 6, resepctively. It is possible to observe some cluster patterns from the distance-based reordering alone. In item (c) of Figure 6, the final clusters for each sample are highlighted in different colours, allowing a visual comparison between the DBS output and the sample distance algorithm.

**Figure 7:**
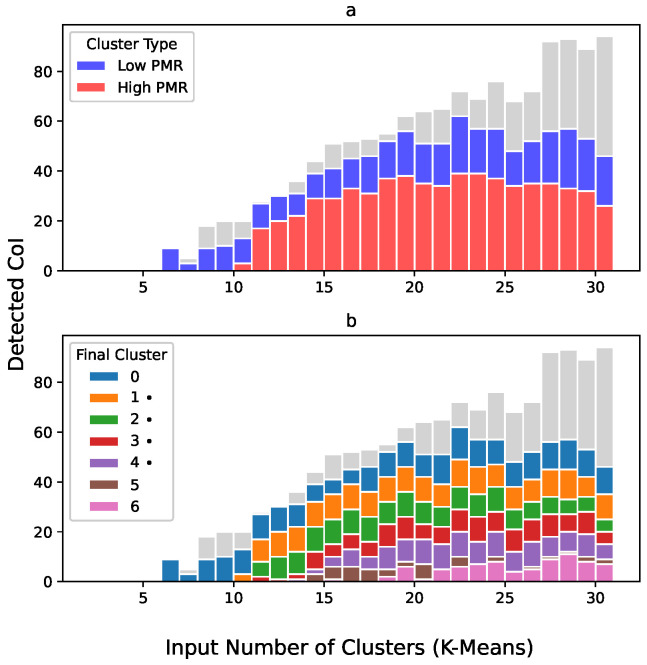
Clusters found per epoch in MkM: (**a**) by cluster type, (**b**) by final cluster. Symbol (•) indicates high PMR cluster.

After applying the $P_{4}$ preprocessing steps, the final clustering was performed by using DBS. DBS found seven final clusters, divided into four clusters with high PMR (PTB Municipality Rate) and three clusters with low PMR. In item (c) of Figure 6, it is shown how some of the rows of the correlation matrix were not selected to any final cluster. In item (a) of Figure 7, it is possible to observe a stagnation or even a reduction in the identification of valid clusters for the highest input number of centres in comparison with median values. In item (b), the same clusters are shown but now separated not by high and low PMR but for individual final clusters.

**Figure 8:**
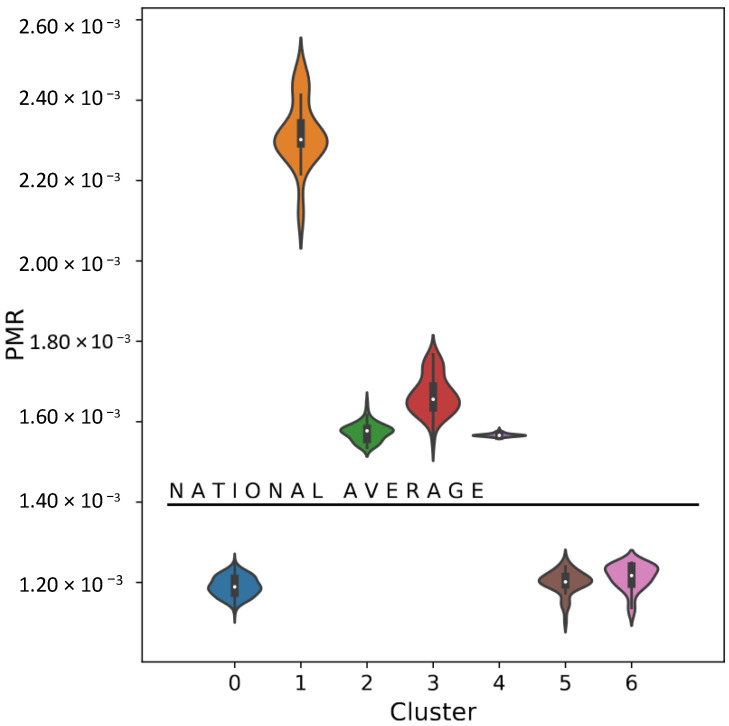
PMR distribution of final clusters.

The CoI’s PMR distribution for each final cluster was calculated. This distribution can be observed in Figure 8, where each cluster is represented on the *x*-axis, the distributions on the *y*-axis and the national average PMR is indicated visually (aprox. $1.4\times 10^{-4}$). It is shown that almost all validated clusters have their centroid PMR varying from $1\times 10^{-4}$ to $2.5\times 10^{-4}$ in comparison to the national average, with the exception of Cluster 1, with a centroid PMR almost $8\times 10^{-4}$ units above the average.

The regional distribution of these clusters was also observed, that is, which municipalities belong to which cluster. Through data visualisation, it is possible to contextualise—as well as to validate—the discovered clusters. Since the input of the problem is social data, it was expected for at least some of the clusters to be located in socially similar concentrated areas. Three visualisations were generated to verify that.

**Figure 9:**
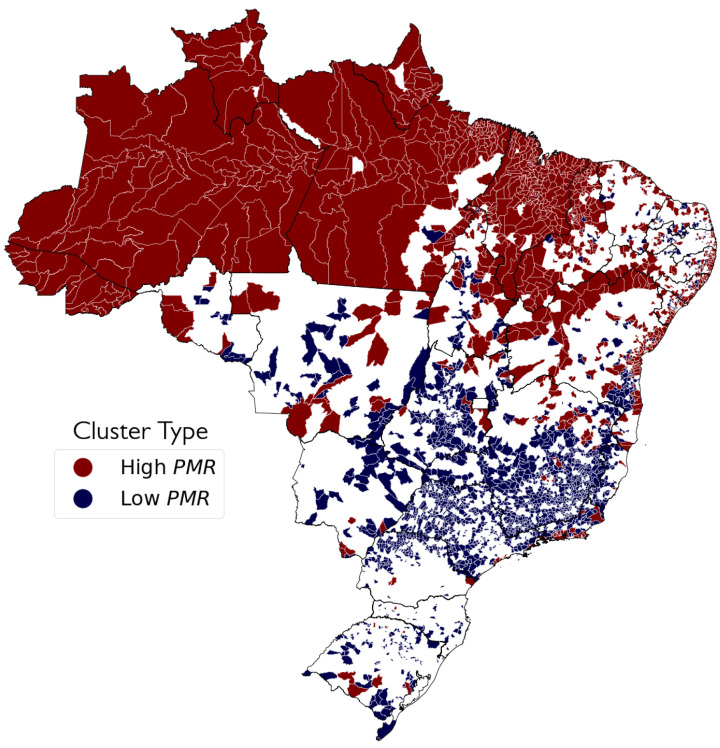
Municipalities by most common type of cluster (high or low PMR). White-coloured municipalities were not classified in a Cluster of Interest.

The first visualisation is shown in Figure 9: it is a binary plot generated using the type of cluster (high or low PMR). The amount of times each municipality was classified into a validated high or low PMR cluster was counted, and each municipality was marked with the type it was mostly classified as. White-coloured municipalities were never classified in a CoI.

**Figure 10:**
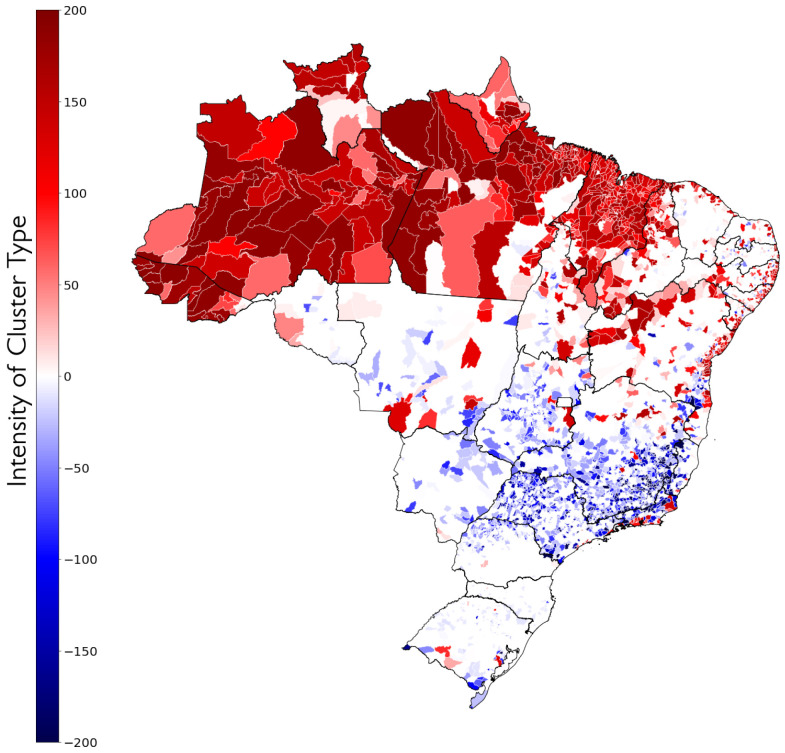
Municipalities by difference of number of times they were classified as each cluster type. Negative values (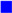
blue) indicate that a municipality was mostly classified in low PMR clusters; positive values (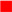
red) indicate it was mostly classified in high PMR clusters.

The second visualisation was generated from the subtraction of the total amount of times in which a municipality was classified as high PMR minus the total amount of times, in which it was classified as low PMR, obtaining a type of degree of intensity or belonging of each municipality to the types of clustering, and it can be seen in Figure 10.

**Figure 11:**
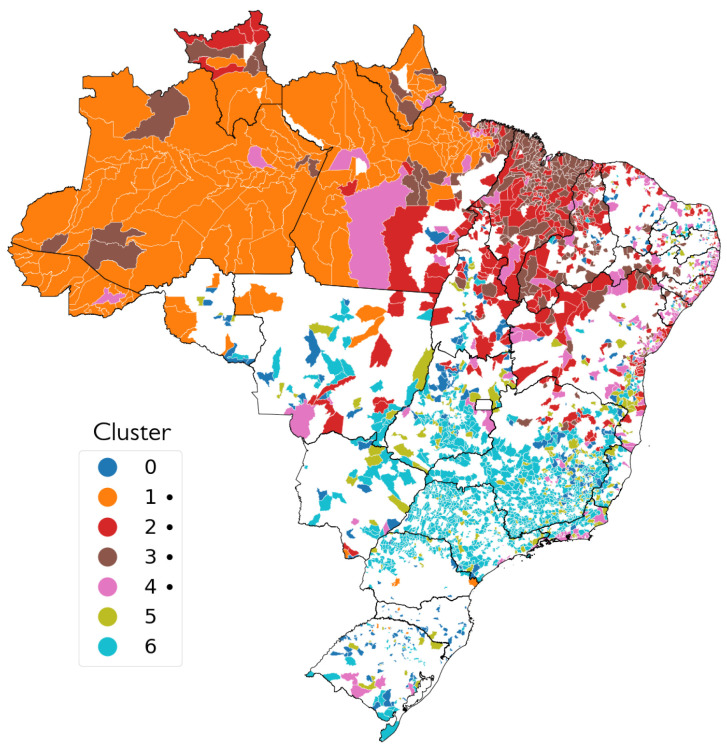
Municipalities by most common cluster. Symbol (•) means high PMR cluster. White-coloured municipalities were not classified in a Cluster of Interest.

The third visualisation, seen in Figure 11, reveals in which of the seven final clusters each municipality was mostly classified by the MkM models, making it possible to visualise the regional aspects of the clusters. Clusters 1, 2, 3, and 6 appear to be more concentrated in specific regions of the map, while 0, 4, and 5 have a more sparse distribution.

Looking at Figure 9 and Figure 10, it is possible to see a clear regional aspect not only for the individual clusters, but also for the types of clusters, with High PMR clusters located mostly in the North and Northeast regions, and the Low PMR clusters in the Centre-South area. In the Northeast, High PMR clusters are concentrated in the state of Maranhão and across the São Francisco River valley. The most intense Low PMR clusters are seen in the state of São Paulo and in Southern Minas Gerais. The North region is almost entirely classified in clusters of High PMR and, as it is shown in Figure 11, the most frequently observed cluster in the region is Cluster 1, notably the one with highest PMR.

In order to measure how the clusters are differing from one another, T-tests were performed to measure the *p*-value of each variable. Two additional sets, treated here as clusters, were created for comparison, *N*, containing all municipalities that were not grouped in any of the final seven clusters, and *A*, containing all municipalities, regardless of clustering.

**Figure 12:**
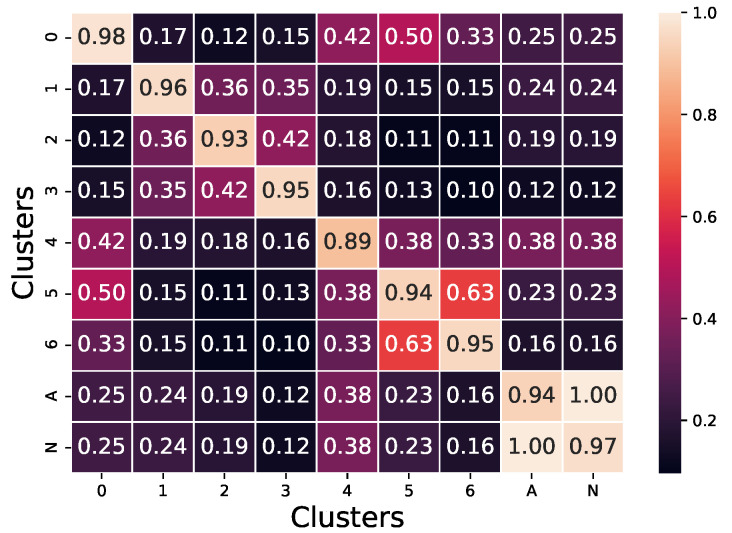
Percentage of variables with a *p*-value under 1% when comparing the pairs of clusters via *t*-test.

The *p*-value was calculated for every variable and for every pair of clusters. The comparison of a cluster to itself was done by generating two random sub-samples of the cluster and testing them against each other. After every *p*-value was determined, the percentage of variables with a *p*-value above the 5% threshold for every pair of cluster was calculated and is shown in Figure 12. It is possible to observe how clusters are significantly distinct from each other through most variables. The closest similarity was observed between clusters 5 and 6 and between clusters 0 and 5 (only 37% and 50% of variables were significantly different, respectively).

**Figure 13:**
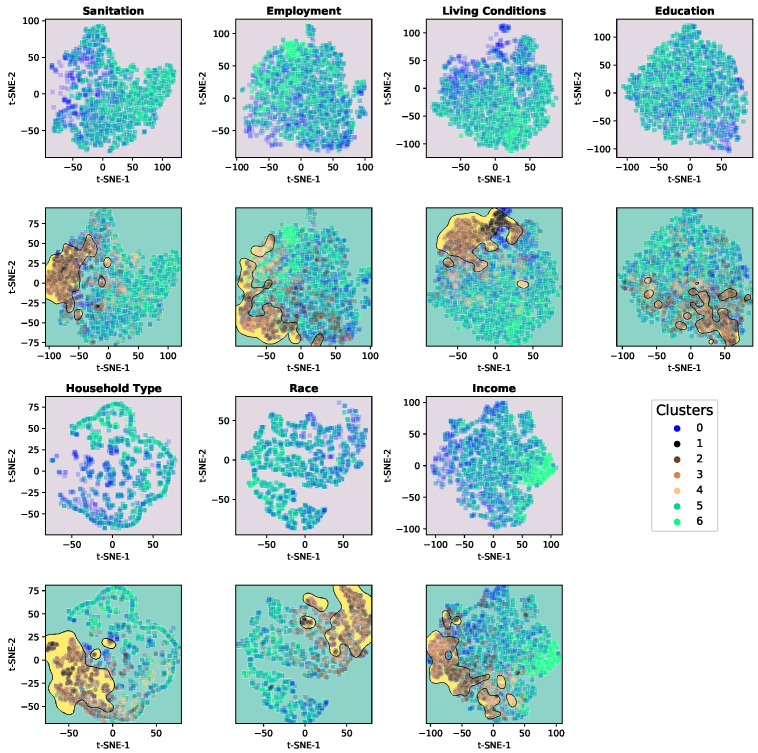
t-SNE 2D representation of final clusters by SES variable type, including SVM-RBF boundaries for type of cluster.

In addition, in order to obtain a general view of how High PMR and Low PMR compare to each other on different aspects of SES, the features used for clustering were categorised into seven segments: Sanitation, Employment, Living Conditions, Education, Household Type, Race and Income. Then, a subset of the data was created for each segment, containing all municipalities, their assigned clusters and only the features of the respective segment. Dimensionality was reduced using t-SNE for visualisation purposes to create 2D maps of the subsets, and an SVM-RBF classifier was applied to the t-SNE maps to find the boundaries in the generated space that best separates High PMR and Low PMR clusters. The t-SNE outcome and the boundaries can be seen in Figure 13. The first (upper) plot for each segment contains only the Low PMR cluster points, the second (lower) also shows the High PMR cluster points and the separation boundaries. It is possible to see, even without reaching the most easily understandable feature-level view, how High and Low PMR clusters follow distinct patterns SES-wise. Some segments, such as Sanitation and Living Conditions, show High PMR cluster points as very well-grouped, and when comparing upper and lower plots, it is almost as if the High PMR points filled an empty space in the lower plot. Others, such as Education and Income, show High and Low PMR cluster points being more mixed up but with High PMR points centred in a smaller area.

Finally, the core of each validated cluster was extracted, containing information about the mean and variance observed for each of the seven clusters. With that information, it is possible to view the detailed features of each cluster.

**Figure 14:**
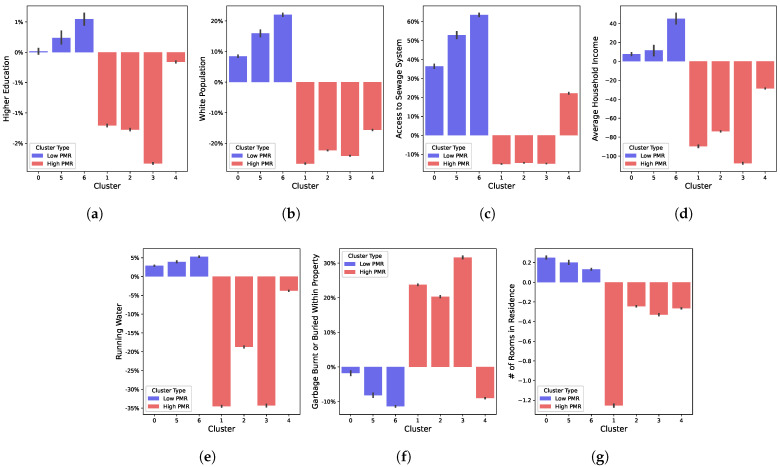
Characteristics associated with clusters: (**a**) Higher Education; (**b**) Race; (**c**) Sewage; (**d**) Income; (**e**) Running Water; (**f**) Garbage Destination; (**g**) Rooms.

It is possible, by checking the individual characteristics of each cluster, to see the relationship between SES factors used as input and the PMR. Figure 14 shows the percentage difference between each cluster and the national average for some of those characteristics: higher education, race, water supply, garbage destination, sewage access and number of rooms in residence. It is noticeable that there is a clear contrast between High PMR and Low PMR clusters among these characteristics.

Cluster 4 is notable for being the only cluster that does not strongly respect this contrast. In Figure 8, Cluster 4 is shown as the one with lowest PMR among those with above-average PMR. In Figure 11, it is visible how cluster 4 is the most disperse among the High-PMR-type clusters, with a noticeable amount of coastal municipalities both in the Northeast and in the state of Rio de Janeiro. In contract, Clusters 1, 2 and 3, with higher PMRs, are concentrated in the North region and in the Northeast region’s countryside.

## 5. Discussion

In this work, unsupervised learning techniques, a two-level clustering, was used two discover clusters of High and Low preterm birth (PTB) rate among Brazilian municipalities while clustering only for SES factors. The clustering resulted in seven final clusters, four with a High PTB Rate and three with a Low PTB Rate, and found significant socioeconomic differences between these High and Low PTB Rate clusters. The results found in this clustering process corroborate and add to the discoveries made by [5]. Their study uses a considerably smaller group for analysis (5297 pregnant women), performs prediction—logistics regression—instead of clustering and uses the SES factors of income, education and employment. Their results suggest that SES factors can help improve accuracy when predicting PTB, thus implying the existence of a relationship between SES factors and PTB. The fact that we were able to observe a similar relationship, with significant difference in PTB among varying SES clusters, even when working with data from a different country with a much larger population and with a larger number of features and a different learning algorithm, strengthens the idea that such a relationship is indeed meaningful. The feature-level analysis endorses the results found by [7,26,27] with High PTB clusters generally having lower levels of income, running water, sanitation access and (mother’s) education.

Medical and health sciences extensively use data, especially biological data, to tackle daily problems. Preterm birth, despite much research, is still not totally comprehended, but studies suggest the influence of external factors, including SES factors. Although deeper research is needed to fully externalise the reasons why SES factors can affect PTB, some strong possibilities are the lack of health assistance/infrastructure leading to worse pregnancy accompaniment by health professionals and, therefore, to higher chances of pregnancy issues leading to PTB, as well as a worse quality of sanitation services causing pregnant women to have overall worse health conditions that could increase the chances of PTB. By finding SES neighbourhoods that are more suitable for the occurrence of PTB, the health system may be able adjust itself better, and earlier, in order to provide assistance to the maximum number of newborns. The use of machine learning clustering techniques allows the analysis of multiple factors at once, with the algorithm naturally adjusting the relevance of each dimension during the training process, creating a situation that is less dependent on a single person’s or a few people’s take on the subject. This characteristic makes it a convenient choice to test and compare assumptions made by less feature-rich models, challenging or reinforcing the current understanding of the subject. In addition, the possibility of applying clustering to the problem also provides a fast, self-adjusting method that could possibly serve as part of a larger, automated and maybe live health management predictive model.

The two-level clustering method described, MkM followed by DBS, allows k-Means clustering in contexts usually not covered by traditional “optimal number of clusters” techniques. By setting a initially designated cluster target rule, k-Means can be used to track down specific sorts of clusters, guaranteeing the significance of the found cluster(s) through recurrence and DBSCAN validation, while also maintaining the algorithm’s explainability factor for posterior analyses. Using this method, we were able to identify seven distinct clusters of notably outlying PTB Rate (10% threshold) as well as how strongly each municipality is associated to those clusters and how different these clusters are amongst each other at the feature level, segment level and overall. The two-level clustering validation behaved as expected, selecting similar cluster centres and discarding the noise generated mostly from the “over-fitting” high number of cluster in some MkM units. The validated final clusters, even if chosen only by their PTB Rate, were shown to be significantly distinguishable in most of the SES factors used in the process. The clustering, working in a PCA-reduced hyperspace, was also able to find clusters that are shown to be distinct, even in specific SES segments such as sanitation and living conditions. In addition, since neighbouring municipalities tend to be more socially alike to each other than to further away municipalities, the more regionally concentrated clusters found here are another previously expected outcome.

Although the goal of finding the High PTB Rate clusters was successful, it represents just a step in what could be followed by a series of analyses concerning each variable or segment of SES individually. Sensible studies and analyses should follow to discover the most relevant features among the 104 considered to explain why such features matter for PTB and to know which features are not relevant—so they can be ignored in a future improved model. Preterm birth analyses reach many areas of study, and SES is just one of the considered factors, so an isolated study such as this is naturally limited in its results. Although many studies have explored the subject analysed here, there was no such study found for comparison that employs precisely the three key points: preterm birth, SES data and unsupervised learning. Finally, this work provides a method that allows cluster analysis on high-dimensional datasets and applies this method to enable the analysis of PTB Rate through SES factors.

## 6. Comparison to State of the Art

The results found in this clustering process corroborate and add to the discoveries made in [5]. Their study uses a considerably smaller group for analysis (5297 pregnant women), performs prediction—logistic regression—instead of clustering and uses SES factors of income, education and employment. Their results suggest that SES factors can help improve accuracy when predicting PTB, thus implying the existence of a relationship between SES factors and PTB. The fact that we were able to observe a similar relationship, with significant difference in PTB among manifold SES clusters, even when working with data from a different country, with a much larger population, with a larger number of features and a different learning algorithm, strengthens the idea that such relationship is indeed meaningful. Their work also notes how such a relationship is restricted; how having such SES information—combined with some individual-level data that they used—is still insufficient for a real-world clinical application of predicting PTB (their work’s goal), which matches with our pre-work thoughts on PTB that it is a multi-factorial problem; and also helps to explain our difficulty and our need to develop an alternative method to achieve our clustering goals using *k*-Means. There are many aspects of PTB invisible to both our work and theirs, and small differences when dealing with different levels of SES are observable but are naturally limited.

How we were able to cluster SES factors in a non-personal level and observes that the regions found had considerable PTB difference draws comparison to and supports the findings in [34]. Their study finds socioeconomic clusters around the city of Paris’ blocks using a spatial clustering technique. They clustered the city into areas and found out the ones where mothers are most likely of experience PTB, and then adjusted the clustering using as control variable an SES index created from 41 original SES variables available. After the adjustment, their results suggest a considerable influence of SES factors on PTB occurrences, as the non-adjusted model showed a much more significant (smaller) *p*-value. This interpretation is supported by the results of our work by the *k*-Means method, as it is perceptible in the Brazilian map of clusters shown for both methods how some regions differ considerably in terms of PTB rate, and similarly to their work, those clusters predominantly assume a regional-centred aspect, creating contiguous areas of similar SES characteristics, of which some have significantly higher or lower PTB rate. Unlike in [34], which uses the geographical location as part of its spatial algorithm, this regional-centred aspect was not intended nor influenced by any location feature, which were removed from the original dataset. For that reason, we obtained an outcome that re-emphasises this strong regional aspect of SES and PTB and, consequently, how neighbouring regions affect the SES and PTB outcomes of a given municipality or city block.

In the feature-wise view presented for *k*-Means method, a few types of variables stood out. Sanitation variables were among the most outstanding in both models, with proper sewage systems, water systems and garbage collecting systems being considerably more present in High PMR regions; this can be linked to and reinforces the findings by [26,39,40]. Although their works do not assess all of these sanitation points, many key variables found are strongly linked to their work, and the “Household with Bathroom” variable, a major point of their analyses, showed a much higher presence on higher PMR clusters, being one of the most diverging features in the *k*-Means method. This was also viewed in a general comparison for the *k*-Means method and corroborates with the findings by [27,41], all of which found statistically significant differences in PTB when stratified by mother’s education. Another clear disparity observed was related to race/skin, with white skin being one of the features most strongly related to Low PMR; this corroborates the findings of the meta-analysis presented by [48], which aggregates several studies related to race and preterm birth occurrences in the United States between 2010 and 2015 and finds a higher risk (1.51 OR) of PTB among historically disfavoured racial groups. Although the exact groups cannot be compared properly, as the Brazilian and U.S. populations have significant racial differences, the high presence of whites in Low PMR clusters and the high presence of *pardos* and indigenous people in the High PMR provide a strong ratification of their results.

## 7. Conclusions

By applying a combination of different clustering methods and dimensionality reduction techniques based on unsupervised learning to socioeconomic data on a municipality-level, it was possible to extract important information on municipal clusters with high and low PTBs. The results obtained here reveal a clear socioeconomic contrast between clusters with high and low risk of PTB, with high-risk clusters predominantly located in regions with the worst social indexes. PTB is a complex and multi-factorial phenomenon and the search for its causes demands analyses of several different aspects. Here, it was seen how the quality of life and public services possibly affect, positively, the reduction in PTB occurrences in such a way that it could and should be included among those aspects.

## Figures and Tables

**Table 1:** SINASC dataset variables, $T_{\mathrm{SN}}$.

SINASC ($T_{\mathrm{SN}}$)	
**Indexer**	Mother’s Residential Municipality Code
**Selected**	Weeks of Pregnancy
**Dropped**	59 others

**Table 2:** CADU Individual dataset variables, $T_{P}$.

CADU Individual ($T_{P}$)	
**Indexer**	ID Individual, ID Household
**Selected**	Gender, Age, Race, Residential Municipality Code, Place of Birth, Disability, Literacy, Type of School, Educational Level, Employment Situation, Type of Job, Total Income, Welfare Income
**Dropped**	Degree of Kinship to Head of Household, Regional Information

**Table 3:** Dataset variables, $T_{F}$.

CADU Household ($T_{F}$)	
**Indexer**	ID Household
**Selected**	Type of Property, Amount of Rooms, Wall Material, Floor Material, Water Supply, Sanitary Drainage, Garbage Collection, Lighting, Pavement, Special Groups Classification, Average Household Income
**Dropped**	Register Date, Modification Date, Update Date, EAS/MS Code, CRAS/CREAS Code

**Table 4:** IBGE dataset variables, $T_{\mathrm{IBGE}}$.

IBGE Population Estimate ($T_{\mathrm{IBGE}}$)	
**Indexer**	Municipality Code
**Selected**	Population
**Dropped**	Municipality Name, Federal Unit, Federal Unit Name

**Table 5:** Summary of datasets used.

Dataset	Dataset	Year	Samples	Features
$T_{\mathrm{SN}}$	SINASC	2018	2,944,932	61
$T_{\mathrm{IBGE}}$	IBGE	2018	5570	5
$T_{P}$	CADU Individual	2018	12,852,599	26
$T_{F}$	CADU Household	2018	4,807,996	23

## Data Availability

Not applicable.
